# Computational and spectroscopic insight into the binding of citral with human transferrin: Targeting neurodegenerative diseases

**DOI:** 10.1016/j.heliyon.2024.e32755

**Published:** 2024-06-08

**Authors:** Anas Shamsi, Moyad Shahwan, Mohammad Furkan, Dharmendra Kumar Yadav, Rizwan Hasan Khan

**Affiliations:** a Centre of Medical and Bio-allied Health Sciences Research, Ajman University, United Arab Emirates; b College of Pharmacy and Health Sciences, Ajman University, United Arab Emirates; c Department of Biochemistry, Aligarh Muslim University, Aligarh, India; d Gachon Institute of Pharmaceutical Science and Department of Pharmacy, College of Pharmacy, Gachon University, Incheon, Republic of Korea; e Interdisciplinary Biotechnology Unit, Aligarh Muslim University, Aligarh, India

**Keywords:** Neurodegenerative diseases, Molecular docking, Spectroscopy

## Abstract

The involvement of neuroinflammation in the pathogenesis of neurodegenerative disorders (NDs) is very significant. Currently, only symptomatic treatments exist, and there are no drugs that modify the progression of Alzheimer's disease (AD) or other NDs. Consequently, there is increasing attention on addressing AD-related neuroinflammation using anti-inflammatory compounds and antioxidants. Currently, there is a growing exploration of dietary phytochemicals as potential therapeutic agents for treating inflammation. Citral, a monoterpene, is under increasing investigation due to its neuroprotective effects. The dysregulation of iron homeostasis is a crucial factor in supporting neuroinflammation, underscoring the significance of proper iron balance. Human transferrin (htf) is a major player involved in iron homeostasis. In this study, we examined binding and dynamics of htf–citral complex through diverse experimental methods. Molecular docking studies revealed that citral binds to crucial residues of htf, forming a stable complex. UV–visible spectroscopy demonstrated binding of citral with htf with good affinity, evident from binding constant of 1.48 × 10^5^ M^−1^. Further, fluorescence spectroscopy entrenched a stable htf-citral complex formation; citral demonstrates an excellent binding affinity to htf with a binding constant of 10^6^ M^−1^. Moreover, fluorescence binding assay at various temperatures deciphered htf-citral complex to be driven by both static and dynamic quenching. The analysis of enthalpy change (Δ*H*) and entropy change (Δ*S*) demonstrated that htf-citral complex formation was driven mainly by hydrophobic interactions.The current work gives a platform to develop innovative therapeutic strategies targeting neuroinflammation through citral, particularly iron homeostasis.

## 1 Introduction

Neurodegenerative disorders (NDs) encompass a range of conditions marked by the gradual degeneration and impairment of the organization and function of the nervous system, predominantly impacting neurons. Typically, these diseases result in a slow deterioration of cognitive abilities, motor functions, and other neurological processes [1]. Various factors contribute to the onset of these conditions, encompassing genetic susceptibility, the accumulation of misfolded proteins, neuroinflammation, and oxidative damages [2,3]. In spite of progress in therapeutics and target identification, “Alzheimer's disease” (AD) remains a prevalent disorder marked by neurons degeneration [4]. According to the Alzheimer's Association, neuronal damage in AD extends to brain regions crucial for fundamental bodily functions like walking and swallowing. AD is a fatal condition, with studies indicating that a significant majority of individuals diagnosed with AD and aged above 65 survive for only a few years [5]. Despite the expanding array of therapeutic and supportive options, it stands as one of the major cause of dementia, impacting millions of people across globe, with expectations of a significant increase in this number in the near future [6,7]. The progressive decline in cognition observed in AD is linked to amyloid-beta (Aβ) and tau proteins accumulation [[8], [9], [10]]. Functional interactions between Aβ protein and tau is linked with neuronal damages and cognitive impairment in AD [11,12]. Thus, the need of the hour is to adopt comprehensive approach to develop potential therapeutic strategies. Aggregation of Aβ occurs in AD resulting in the formation of “extracellular plaques”, thereby impacting variety of cellular functions [8], namely, disrupting calcium signaling and generating “reactive oxygen species” (ROS) and “reactive nitrogen species” (RNS) [13,14]. Moreover, Aβ accumulation also triggers inflammation, initiating microglia activation around the plaques [15]. Neurofibrillary tangles (NFTs), are the second significant AD pathological hallmarks and are attributed to tau hyperphosphorylation, resulting in microtubules destabilization [16]. Neuroinflammation stands out as a significant AD pathological component [17]; many studies reporting links between neuroinflammation and AD pathogenesis [[18], [19], [20]].

Unbound iron serves as a potent neurotoxin, leading to the production of ROS and inducing oxidative stress. In its free state, iron (Fe) acts as a robust neurotoxin capable of triggering oxidative stress, a pivotal pathological characteristic in neurodegeneration. Oxidative stress is instigated through a process wherein reactive radicals, such as hydroxyl radicals (-OH), are produced. This process ultimately causes damage to the cells, which results in cell death, which is the most common cause of neuronal loss in AD. The production of ROS and RNS, in the inflammatory process, influences Fe homeostasis by interacting with “iron-regulatory proteins” (IRPs) [21]. The onset for neuroinflammation in NDs is not completely understood; however, microglia and astrocytes activation ensure a strong inflammatory response. The proteins, human transferrin (htf) and ferritin serve as major players in Fe homeostasis. htf, amongst most abundant serum protein in plasma, is responsible for transport of both endogenous and exogenous compounds [22]. The single-chain glycoprotein has 679 amino acids [23] and has two metal binding sites with approximately 30 % iron saturation [24] and serves as a vital Fe-transporter and thus it is associated with Fe-transferrin receptor, and the cells' endosomal compartment [25,26] and has various physiological and pharmacological functions and thus is amongst the most extensively studied proteins along with human serum albumin (HSA) [27]. The pro-inflammatory cytokines alter the Fe-related proteins responsible for iron homeostasis, resulting in deposition of Fe in neurons. Fe loading in brain regions in AD is a consequence of earl neuroinflammation. The release of ROS, RNS and cytokines from glial modulates the activity of Fe-proteins crucial for Fe homeostasis. These findings underscore the significance of Fe-homeostasis in neuroinflammation associated with AD, highlighting the crucial role of htf in AD therapeutics.

In recent times, researchers are focusing to target AD-related inflammation with compounds exhibiting anti-inflammatory and antioxidant properties [[28], [29], [30]]. The involvement of natural compounds in AD has garnered increasing interest and research attention. Various natural compounds, sourced from plants or other origins, have demonstrated potential neuroprotective effects and could potentially influence the course of AD [31]. Citral, the primary component of lemongrass, is predominantly present in oils from various plant species such as *Lemon myrtle* and *Listea citrata*. Known for its robust lemoncent and essence, citral is commonly used as a dietary additive [32]. It possesses antibacterial properties [33], attributed to the generation of ROS in microorganisms [34]. Additionally, citral has demonstrated antioxidant activity and enhances the expression of “Brain-Derived Neurotrophic Factor” (BDNF) [35,36].

Keeping in view the importance of Fe-homeostasis in neuroinflammation associated with AD and significance of phytochemicals in targeting this AD-related inflammation, our study aims to decipher the binding mechanism of citral with htf, major protein involved in Fe-homeostasis using a comprehensive computational and experimental approaches.

## 2 Material and methods

### 2.1 Materials

Human transferrin (htf) and citral were purchased from “Sigma Aldrich” (St. Louis, USA). We created a stock solution of htf at a concentration of 5 mg/ml and subsequently diluted it to the working concentration for use in experiments. All remaining chemicals utilized were of the highest analytical grade. All the experiments have been performed in triplicates and mean of the values are considered for final calculations.

### 2.2 In silico toxico-kinetic predictions

We employed web-based services, namely, SwissADME”” (http://www.swissadme.ch) [37], “pkCSM” (https://biosig.lab.uq.edu.au/pkcsm) [38], and “ProTox” (https://tox-new.charite.de/protox_II) [39] to decipher the toxicokinetic properties of citral. The chemical structures of citral in smiles format were used as inputs to execute the predictions by default settings.

### 2.3 Molecular docking

We conducted molecular docking utilizing InstaDock software [40] version 10. htf structure was retrieved from the Protein Data Bank (PDB ID: 3V83), due to its high resolution of 2.10 Å, while citral structure was obtained from PubChem (PubChem CID 638011). We used a blind search space approach, encompassing the entire protein structure to ensure a thorough exploration of potential binding sites. All remaining docking parameters were maintained at their default settings, consistent with those employed in previously published reports. Various other tools, namely, PyMOL and Discovery studio [41] were used on a Windows 11-based DELL® workstation to have an in-depth analysis of this interaction between citral and Htf.

### 2.4 UV–vis absorbance

The absorbance spectrum of htf was measured at 4 μM. The spectra of the protein with increasing concentration of the ligand were also measured (Shimadzu UV‐1700, path length 1 cm). The protein was titrated with citral from 0 to 8 μM concentrations. The reading range was kept 240 nm–340 nm. The binding constant (*K*) was calculated using equation (1):

(1) $$\frac{Aο}{\;Aο-A}=\frac{\varepsilon \text{Htf}}{\varepsilon B}+\frac{\varepsilon \text{Htf}}{\varepsilon BK}X\frac{1}{C}$$

### 2.5 Steady-state fluorescence studies

To assess the binding strength between citral and htf, we conducted a fluorescence-based quenching experiment at different temperatures using a Jasco spectrofluorometer (FP-6200). Our analysis followed established protocols from previous studies [21,42,43]. In a bid to ensure accuracy, we corrected “inner filter effect”. Herein, htf was excited at 280 nm to capture its overall intrinsic fluorescence, and spectra was recorded in the range of 300–400 nm. The concentration of htf was fixed at 4 μM while citral was varied in the range of 0–8 μM. The obtained data was subjected to Stern-Volmer (SV) and modified Stern−Volmer (MSV) equations to estimate various parameters that characterize the strength of the htf-citral interaction and to elucidate the type of quenching, as per earlier reports [44,45].

### 2.6 Thermodynamics of the complex

van't Hoff equation (Equation (2)) [45] is useful in studying how changes in temperature impacts chemical equilibria and further provide insights into the thermodynamic behaviour of chemical reactions, revealing multiple thermodynamic parameters linked to the htf-citral complex.

(2) ΔG=−RTLnK=ΔH−TΔS

### 2.7 Synchronous fluorescence

In this experiment, 4 μM htf was subjected to titration using various concentrations of citral (0–10 μM). During these measurements, protein was excited at 240 nm, and the emission spectra were recorded within the range of 255 nm–600 nm, thereby maintaining a constant Δ*λ* (wavelength difference) of 15 nm for detecting tyrosine residues. Additionally, for the tryptophan residues, a Δ*λ* of 60 nm was consistently applied, with the excitation wavelength fixed at 240 nm, and emission data collected within the 300 nm–600 nm.

## 3 Result and discussions

### 3.1 Toxico-kinetics predictions

Toxicokinetic properties are the characteristics and processes that administer the behaviour of toxins in an organism's body. The properties include absorption, distribution, metabolism, and elimination (ADME) of the compound and its toxicological effects. Toxicokinetic properties of citral were predicted *in silico* using, “SwissADME”, “pkCSM”, and “ProTox” tools, and the estimated ADMET properties are summarized in Table 1. Citral has a small molecular structure and weighs 152.23 g/mol, with very less polar surface area and a high lipophilic area. Citral is likely lipophilic and typically poorly soluble in water, but easily dissolve in nonpolar solvents such as organic solvents, oils, and lipids. Lipophilic molecules interact favourably with nonpolar substances and are more likely to accumulate in the lipid compartments of the body. The lipophilic nature of citral makes it readily available for absorption as it can easily diffuse through the cell membrane and cross the biological barriers as compared to hydrophilic compounds. The prediction results show citral has a high absorption rate through the gastrointestinal tract and thus it crosses “blood-brain-barrier” (BBB). However, the compound has a very low skin permeability of −5.08 cm/s. The predicted logBB values of citral of 0.626 predicted that the compound has a very high probability to reach the brain and interact with the proteins in the brain. The 50 % lethal doses (LD_50_) via the oral route were reported to be 500 mg/kg. In the context of chemical safety and risk assessment, substances with high LD50 values are generally considered to have lower acute toxicity and may be considered less hazardous. Citral is considered to have no toxic effect and is placed in category 4 of toxicity as predicted by ProTox tool. The compound does not exhibit any carcinogenic, mutagenic or immunogenic effect with moderate doses.

**Table 1:** ADMET properties of CL obtained from SwissADME, pkCSM, and ProTox.

ADMET	CL
MW (g/mol)	152.23 g/mol
Number of hydrogen bond acceptors	1
Number of hydrogen bond donors	0
Topological Polar Surface Area (Å^2^)	17.07 Å^2^
Water solubility (Log S)	‒2.3
Octanol/water partition coefficient (log P_o/w_)	2.47
GI absorption (% Absorbed)	High (95.317)
Skin permeability (logKp)	High (−2.41)
Fraction unbound (Fu)	0.42
BBB permeability (log BB)	Yes (0.626)
CNS permeability (log PS)	‒1.986
CYP2D6 substrate	No
CYP3A4 substrate	No
Total clearance (log mL/min/kg)	0.376
Immunotoxicity	No
Hepatotoxicity	Inactive
Carcinogenicity	Inactive
Immunotoxicity	Inactive
Mutagenicity	Inactive
Cytotoxicity	Inactive
Skin sensitization	Inactive
Predicted LD_50_ (mg/kg)	500

### 3.2 Molecular docking

**Fig. 1:**
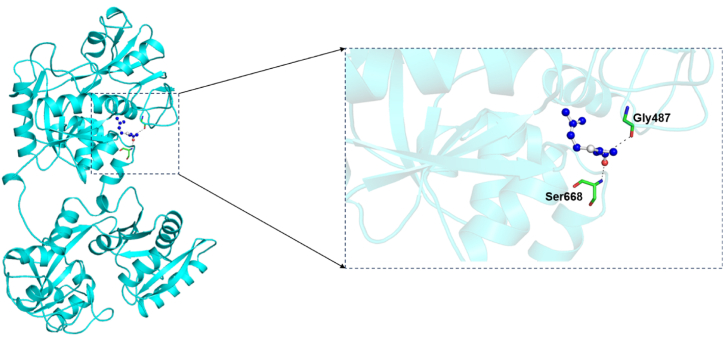
Cartoon representation showing the docked citral interacting with htf. Zoomed view of the binding pocket showing the residues involved in hydrogen bonding.

**Fig. 2:**
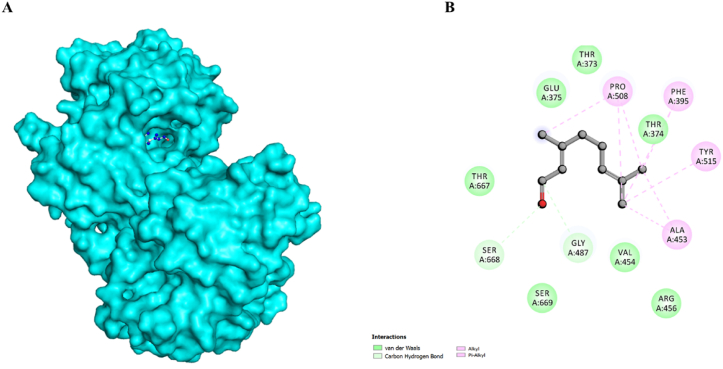
**(A)** Surface view of htf with citral in the binding pocket depicted as ball and sticks. **(B)** 2-D structural representation of htf residues engaged in interactions with citral.

Molecular docking was conducted to identify the key residues driving the htf-citral complex formation, thereby illuminating binding mechanism of citral with htf. Citral presented a binding affinity value of −5.0 kcal/mol and a pKi value of 3.67 along with a noteworthy “ligand efficiency value” of 0.4273 kcal/mol/non-H atom -. The docked conformers of citral were investigated to decipher the potential binding interactions with crucial residues of htf. Fig. 1 shows that citral occupied a deep position in the binding pocket, forming various interactions with the several key binding pocket residues. Fig. 2A shows htf represented as surface with citral occupying a position in the binding pocket. Fig. 2B shows 2-D structural representation of htf residues interacting with citral. It is noteworthy to mention that citral formed two carbon hydrogen bonds with Gly487 and Ser668 and moreover, we observed other vital interactions, including “Alkyl”, “π-Alkyl”, and “van der Waals forces”, with multiple residues of htf, which stabilized the complex.

### 3.3 UV–vis absorbance

**Fig. 3:**
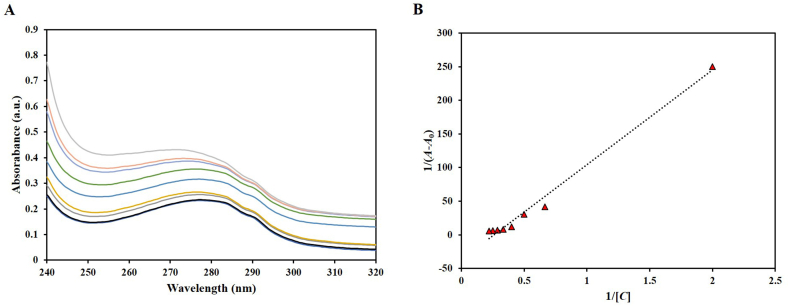
**(A)** UV–visible spectra of native htf and in the presence of citral. (B) “Double reciprocal plot” of htf-citral complex.

UV–vis spectroscopy is commonly utilized for the examination of ligand-protein binding [46]. When ligand binds to the protein resulting in protein-ligand complex formation, it is accompanied with changes in absorbance and/or shift of the spectrum.; these changes are measured. htf concentration was static at 4 μM while ligand, citral, was varied in a dose dependent manner and an evident hyperchromism was observed with subtle changes in peak position (Fig. 3A). The data was fitted into the “double reciprocal plot” of 1/(*A* − *A*_0_) v/s 1/*C* (Fig. 3B). We computed the binding constant (*K*) for the htf-citral complex from the ratio of the intercept and the slope of this plot (Fig. 3B). The determined *K* value of 1.48 × 10^5^ M^−1^ indicates that citral binds to htf with a strong affinity, forming a stable complex.

### 3.4 Steady state fluorescence

**Fig. 4:**
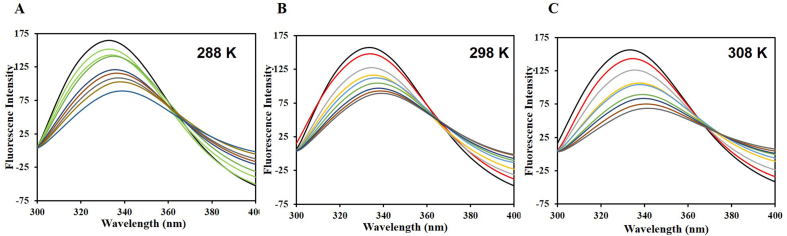
Steady state fluorescence of htf in the absence and presence of CL (0–8 μM) at **(A)** 288 K, **(B)** 298 K and **(C)** 301 K.

**Fig. 5:**
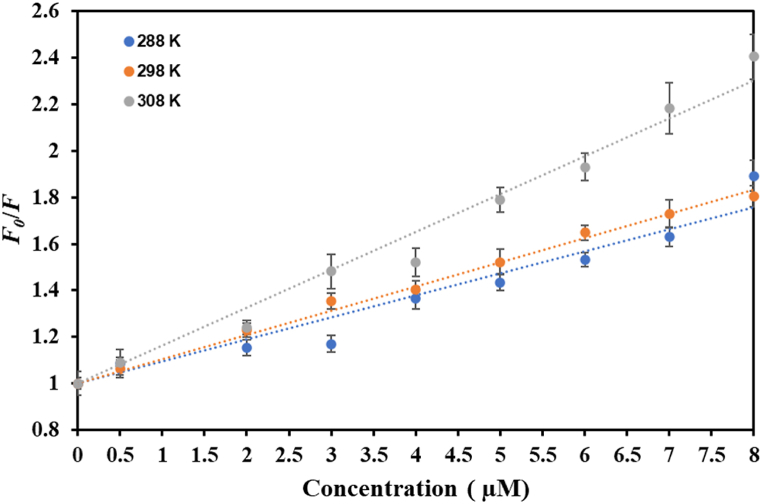
SV plots of htf-citral complex at three different temperatures.

The fluorescence-based binding assay stands out amongst top notch to study protein-ligand interactions. This method proves instrumental to characterize protein-ligand complex as it aids in estimation of various binding parameters of the complex. Primarily, there are three intrinsic fluorophores: tyrosine (Tyr), tryptophan (Trp) and phenylalanine (Phe), with Trp contributing maximal in the intrinsic fluorescence. Fluorescence spectroscopy aids in the calculation of various binding parameters in a way that the surrounding environment is altered around these intrinsic fluorophores after ligand binds and this corresponds to alterations in the fluorescence spectra that are recorded. Native htf shows fluorescence maximum at around 334 nm, upon exciting it at 280 nm, exhibiting the intrinsic characteristics of intrinsic fluorophores [47]. Citral quenched the intrinsic fluorescence of htf in a in a manner dependent on the dosage, keeping htf static at 4 μM. Moreover, an isobestic point was observed at around 367 nm at all the three temperatures (Fig. 4), implying the formation of complex between citral and htf [48]. The existence of the isobestic point also shows that citral and htf have reached an equilibrium, resulting in the formation of a 1:1 complex. A similar quenching pattern was observed at all three temperatures (288 K, 298 K and 308 K) (Fig. 4A–C). Fluorescence quenching can be “static”, “dynamic” or a “combination of both” [49,50]. The fluctuation of binding parameters with temperature reveals the type of quenching that propels the formation of the complex, thus highlighting the importance of quenching at various temperatures. The obtained quenching data was fitted into SV equation that resulted in generation of SV plot at different temperatures (Fig. 5); Stern-Volmer constant (*K*_sv_) computed from the slope of these plots. The variation of *K*_sv_ sheds light on the type of quenching driving the complex formation.

Different parameters obtained from SV equation at various temperatures are illustrated in Table 2. In the static quenching, a decrease in *K*_sv_ values while in the dynamic quenching an increase in *K*_sv_ values is observed with a corresponding increase in temperature. We found (Table 2) that with increasing temperatures, a corresponding increase in *K*_sv_ was observed, implicative of the fact that dynamic quenching governs the htf-citral complex formation [44]. Moreover, the value of “bimolecular quenching rate constant” (*K*_q_) was estimated employing another equation:

$$K_{q}=\frac{K_{sv}}{\tau_{0}}$$

**Table 2:** Binding parameters obtained from Stern-Volmer equation.

Temperature, K	*K*_*sv*_ (10^4^ M^−1^)	*K*_q_ (10^13^ M^−1^ s^−1^)
288	9.48	1.64
298	10.43	1.80
308	16.26	2.81

The estimated *K*_q_ values deciphers the mode of quenching; for htf-citral complex, the values were substantially higher than the “maximum dynamic quenching constant” (∼10^10^ M^−1^ s^−1^) [45], signifying that static quenching drives the htf-citral formation. Thus, from above observations it can be concluded that htf-citral complex formation is driven by a combination of static and dynamic quenching.

**Fig. 6:**
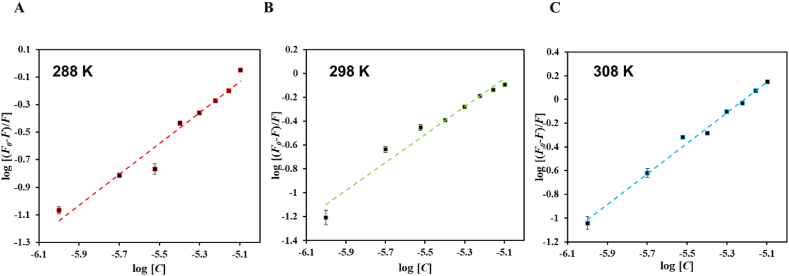
MSV plots of htf-citral complex at three different temperatures.

Further, the data was fitted into MSV equation to compute *K* and number of binding sites (*n*) that demonstrates strength of binding of ligand with the protein. The obtained data at different temperatures generated three different MSV plots (Fig. 6); where the intercept of the plot gives *K* and the slope corresponds to *n*. Table 3 shows *K* and *n* computed at various temperatures and *K* values obtained clearly signifies that citral binds to htf with an excellent affinity at lower temperatures while at higher temperatures citral shows good affinity towards htf i.e. comparatively a more stable complex is formed at low temperatures, though significant affinity is shown towards htf even at higher temperatures. Altogether, the observations depict that citral shows a significant affinity towards htf and forms a stable complex which is governed by a combination of static and dynamic quenching.

**Table 3:** Binding and thermodynamic parameters obtained from MSV plots.

Temperature (K)	*K* 10^6^M^−1^	Δ*G* kcal mol^−1^	Δ*S* cal mol^−1^K^−1^	Δ*H* kcal mol^−1^	TΔS kcal mol^−1^
288	0.37	−7.23151	105.73	−23.22	30.45201
298	0.82	−8.28888			31.50938
308	5.34	−9.34624			32.56674

### 3.5 Thermodynamics of the complex

**Fig. 7:**
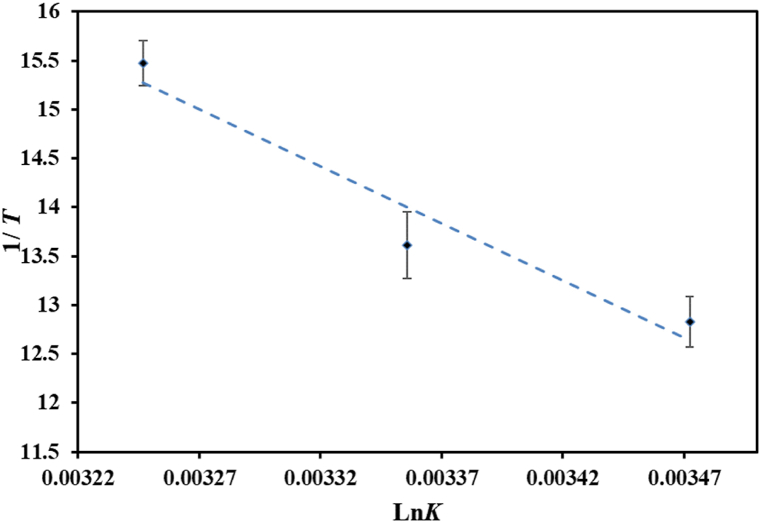
van't Hoff plot of htf-citral complex.

The binding mechanism of citral with htf was ascertained using fluorescence spectroscopy at different temperatures. After this, the aim was to delineate thermodynamic parameters using the “van't Hoff equation”. Fig. 7 demonstrates “van't Hoff plot” for htf-citral complex, slope of it equals to the value of -*ΔH*/R, while intercept equivalent to *ΔS*/R. As per the findings by Ross and Subramaniam [51], the presence of negative enthalpy (*ΔH*) and entropy (*ΔS*) values in a reaction implies that major contribution is from “van der Waals forces” and/or “hydrogen bonding”. Conversely, positive values of *ΔH* and *ΔS* suggests that “hydrophobic interactions” are predominant. More specifically, a condition with negative *ΔH* and positive Δ*S* values indicates that “electrostatic interactions” play a driving force in the reaction. We obtained positive values of *ΔH* and *ΔS,* thereby revealing that “electrostatic interactions” primarily drive the complex formation. Table 3 illustrates the thermodynamic parameters associated with the interaction between htf and citral. Moreover, a negative *ΔG* was obtained, implying the binding of citral with htf to be spontaneous in nature.

### 3.6 Synchronous fluorescence

**Fig. 8:**
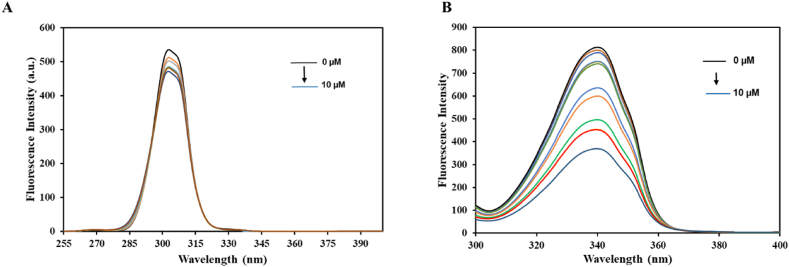
Synchronous fluorescence spectra of htf in the absence and presence of citral for **(A)** Tyrosine (Δλ = 15 nm) and **(B)** Tryptophan (Δλ = 60 nm).

In a bid to have a more comprehensive insight into the impact of the ligand on the microenvironment of the fluorophore, we carried out synchronous fluorescence experiments. The maximum emission (*λ*_max_) of the protein aligns with alterations in polarity surrounding the fluorophore molecule. Maintaining a constant difference in Δ*λ*(*λ*_em_ − *λ*_ex_), the spectra delineate distinct features characterizing the microenvironment of the “aromatic amino acids”, specifically Trp and Tyr [52]. Fig. 8 illustrates the synchronous fluorescence spectra of native htf and with the citral presence. Spectra corresponding to Tyr residues (Δ*λ* = 15 nm*)* is presented in Fig. 8A while Fig. 8B represents the synchronous emission spectra of for tryptophan (Δ*λ* = 15 nm). It is apparent that a slight shift was observed on maximum emission wavelength of try residues implying that subtle changes occurred in the local environment around these residues. There was no variation in the maximum emission wavelength of Trp residues, however there were significant changes in the fluorescence intensity, implying occurrence of changes in the local environment around Trp residue.

## 4 Conclusion

We employed a blend of spectroscopic and computational approaches to shed a light on the interactions between htf and naturally occurring phytochemical, citral. Molecular docking played an important role in elucidating the specific residues driving the complex formation. A detailed analysis of the interactions revealed formation of several hydrogen bonds and other key interactions and also indicative of the fact that citral occupies htf's binding pocket. Further, UV visible spectroscopy showed a strong binding affinity between citral and htf. This strong binding was further ascertained by fluorescence spectroscopy that established that citral binds to htf with an excellent affinity forming a stable htf-citral complex. Fluorescence binding assay at distinct temperatures revealed the complex formation to be driven by “combination of static and dynamic quenching”. In summary, this study revealed the binding affinity and stability of htf-citral complex. These insights give a solid groundwork for future research in lieu of therapeutic potential of citral in the treatment of AD, particularly within the context of iron homeostasis.

## Data availability statement

Data included in article/supplementary material/referenced in article.

## CRediT authorship contribution statement

**Anas Shamsi:** Writing – original draft, Validation, Supervision, Methodology, Investigation, Funding acquisition, Formal analysis, Data curation, Conceptualization. **Moyad Shahwan:** Software, Methodology, Data curation. **Mohammad Furkan:** Writing – original draft, Methodology, Investigation, Formal analysis, Data curation. **Dharmendra Kumar Yadav:** Writing – review & editing, Methodology, Investigation, Funding acquisition. **Rizwan Hasan Khan:** Writing – original draft, Visualization, Supervision, Investigation, Formal analysis, Data curation, Conceptualization.

## Declaration of competing interest

The authors declare that they have no known competing financial interests or personal relationships that could have appeared to influence the work reported in this paper.
